# Encoding order and developmental dyslexia: A family of skills predicting different orthographic components

**DOI:** 10.1080/17470218.2014.938666

**Published:** 2015-01-01

**Authors:** Cristina Romani, Effie Tsouknida, Andrew Olson

**Affiliations:** 1 School of Health and Life Sciences, Aston University, Birmingham, UK; 2 School of Psychology, University of Birmingham, Birmingham, UK

**Keywords:** Developmental dyslexia, Serial order, Sequential presentation, Visual impairments, Lexical learning

## Abstract

We investigated order encoding in developmental dyslexia using a task that presented nonalphanumeric visual characters either simultaneously or sequentially—to tap spatial and temporal order encoding, respectively—and asked participants to reproduce their order. Dyslexic participants performed poorly in the sequential condition, but normally in the simultaneous condition, except for positions most susceptible to interference. These results are novel in demonstrating a selective difficulty with temporal order encoding in a dyslexic group. We also tested the associations between our order reconstruction tasks and: (a) lexical learning and phonological tasks; and (b) different reading and spelling tasks. Correlations were extensive when the whole group of participants was considered together. When dyslexics and controls were considered separately, different patterns of association emerged between orthographic tasks on the one side and tasks tapping order encoding, phonological processing, and written learning on the other. These results indicate that different skills support different aspects of orthographic processing and are impaired to different degrees in individuals with dyslexia. Therefore, developmental dyslexia is not caused by a single impairment, but by a family of deficits loosely related to difficulties with order. Understanding the contribution of these different deficits will be crucial to deepen our understanding of this disorder.

The idea that dyslexics have a special difficulty in the processing of serial order is often expressed by parents and teachers who report that dyslexic children have trouble in learning the days of the week and the months of the year and make errors of reversal and misordering in spelling (Kaufman, 1980; Terepocki, Kruck, & Willows, 2002). In addition, a number of impairments characterizing dyslexic participants can, in principle, be attributed to difficulties in processing order (see below). In spite of this, only a few studies have directly investigated order-encoding difficulties in dyslexia. One exception is a recent study by Szmalec, Loncke, Page, and Duyck (2011), which investigated the ability to learn sequences of visual letters, auditory letters, or spatial locations (represented by dots on a computer screen) with a Hebb paradigm. This paradigm intermixes sequences presented only once (filler sequences) with sequences that are repeated a number of times (Hebb sequences), so that one can measure learning across presentations. The performance of dyslexic participants was equivalent to that of controls for the filler sequences, but impaired for the Hebb sequences, suggesting that a selective deficit in *serial order learning* can be the source of reading difficulties in dyslexia. The strength of this conclusion, however, is limited by a number of considerations.

It is unclear whether serial order deficits are really limited to *learning* in dyslexia as suggested by Szmalec et al. (2011). Dyslexics are often found to be impaired in tasks involving the encoding and immediate recall of order information both in the auditory and in the visual modality (see below for an extensive review). Therefore, the lack of differences with the control group reported in the filler condition is puzzling and could reflect a floor effect rather than normal performance. Secondly, it is unclear how the order deficit identified with a Hebb learning paradigm relates to other possible order deficits identified with other tasks. Finally and crucially, it is unclear how performance on this task relates to reading and spelling. If serial order deficits are a crucial component of developmental dyslexia, they should explain variation in reading and spelling performance in dyslexic and/or control participants. The purpose of the present study is to build on Szmalec's study to learn more about the relation between encoding order and orthographic learning in dyslexia.

First of all, we contrast different modalities of order encoding. Within the same task, we compare conditions that tap spatial and temporal order encoding. In addition, we contrast this task with a lexical learning task that has a strong component related to long-term, abstract encoding of order. Secondly, we assess the associations between these order-encoding tasks and reading and spelling tasks. Exploring these associations is crucial to understanding whether the difficulties of the dyslexics reflect a single deficit in representing and encoding order or, rather, whether order is encoded through different, partially independent skills (involving phonological processing, spatial attention, temporal order encoding, and learning of abstract order), which support reading and spelling in different ways.

## The task

We used an order-reconstruction task that measured encoding of order without a learning component and without involving linguistic representations that overlap with reading and spelling tasks (we used series of Hindi and Japanese characters, unfamiliar to our participants; thus, from now on, the H&J task). We contrasted a simultaneous condition, where the characters of the series to reconstruct were presented together, in a line, and a sequential condition, where the characters were presented one at a time at fixation. The first condition taps *spatial order* because order is represented through the spatial relationship between the different characters. The second condition taps *temporal order* because each of the visual characters must be associated with a given point in time if the order of the sequence is to be reconstructed at a later point. Finally, we interspersed conditions involving order reconstruction with conditions involving recognition of individual characters without an ordering component.

A similar task was previously used in a single case study by Romani, Ward, and Olson (1999). A.W. was a young dyslexic adult with normal phonological skills, but severely impaired written learning, word spelling, and nonword reading. In addition, A.W. was impaired in the H&J order reconstruction task when the characters were presented sequentially, but not when they were presented simultaneously, in a line. This was attributed to A.W.'s exceptionally good visuospatial memory, which may have allowed him to encode spatial order in spite of difficulties with temporal sequences. The present study will verify whether selective problems with temporal order occur commonly in adults with developmental dyslexia across people with different levels of visuospatial ability. Finding no difficulties with spatial order would be consistent with the fact that developmental dyslexics generally have normal visual memory (see also Hawelka & Wimmer, 2008; Shovman & Ahissar, 2006; Von Karolyi, Winner, Gray, & Sherman, 2003), and difficulties occur only when fine allocation of attention is required as in processing closely spaced arrays (see later for a review). Two previous studies have used a version of the H&J task to assess association with reading and spelling in college students with different levels of abilities and found inconsistent associations with spelling (Holmes, Malone, & Renenback, 2008) and weak associations with reading (Holmes, 2006). These associations, however, can be stronger when groups of dyslexics are involved.

## Relation with other impairments

We believe it is important not only to demonstrate an independent impairment, but also to verify associations between different tasks that require encoding of order, as well as between these tasks and a range of orthographic tasks. A fact that is often overlooked is that developmental dyslexics are not homogeneously impaired across tasks. Some individual have more difficulties with reading, others with spelling; equally some individuals have selective difficulties with some stimuli and not others (e.g., words vs. nonwords). These difficulties, in turn, may be caused by different underlying cognitive weaknesses (Di Betta & Romani, 2006; Menghini et al., 2010; Romani, Di Betta, Tsouknida, & Olson, 2008). Patterns of associations between tasks also offer a special tool to understand how different difficulties in processing order can contribute to developmental dyslexia. Different hypotheses make different predictions. The hypothesis that *a single deficit of temporal order is central to dyslexia* predicts extensive associations among tasks tapping order and between these tasks and reading and spelling tasks (from now on, orthographic tasks). The hypothesis that order deficits are not a cause of dyslexia, but only a *marker,* predicts limited or no correlations. Deficits may co-occur because their neurological bases happen to be impaired together in development, but there is no reason to expect a close correspondence in the severity of impairment across tasks. Finally, the hypothesis that there is *a family of deficits* that relate to order encoding, but involve independent skills, predicts that while different tasks tapping order processing may be intercorrelated, they will show different patterns of association with orthographic tasks.

Before moving to our experimental investigation, we want to analyse in more detail the relation between different skills related to order encoding. Both temporal and spatial order encoding are closely related to other skills, which, although supporting encoding of order, are not identical to it (i.e., phonological processing and temporal resolution support encoding of temporal order; visual attention supports encoding of spatial order). With our paradigm we want to distinguish “proper” order deficits from deficits in these other supporting skills. Finally, it is important to distinguish deficits in encoding temporal and spatial order from representational deficits where order is represented in an abstract fashion, without any direct reference to time or spatial positions.

## Temporal order, phonological processing, and temporal resolution

Probably the most successful single-cause explanation of developmental dyslexia is in terms of difficulties in phonological processing (for reviews see Castles & Coltheart, 2004; Snowling, 2000). Thus, any demonstration of order difficulties needs to distinguish them from difficulties in phonological processing. However, the relation between order encoding and phonological tasks is not clear. On the one hand, dyslexics may have difficulties with phonological tasks because they are impaired in encoding temporal order. Span tasks require remembering the order of words. Nonword repetition and tasks tapping phonological awareness (phoneme counting, phoneme deletion, spoonerisms, etc.) involve remembering the order of phonemes within words. On the other hand, the causal link could be reversed. Dyslexics may have difficulties with tasks involving temporal order because of phonological difficulties. Phonological representations greatly support order encoding because phonemes unfold in time, and their order is subject to articulatory constraints. If this hypothesis is correct, however, dyslexics should have no difficulties in tasks that involve the ordering of representations that are not phonological or not easily converted into a phonological representation. To minimize the contribution of phonological representations, our paradigm involves visual/nonverbal stimuli that are not easily nameable.

A second concern is to distinguish difficulties with temporal order from possible difficulties in temporal resolution. If two events cannot be distinguished in time, they cannot be ordered. Difficulties with temporal resolution have been hypothesized to arise as a consequence of magnocellular impairments. They would affect processing sequences of rapidly presented stimuli, auditory or visual. A temporal window that is too wide increases stimulus persistence and creates difficulties in distinguishing one stimulus from the next (e.g., Hansen, Stein, Orde, Winter, & Talcott, 2001; Stein, 2003; for auditory stimuli see Farmer & Klein, 1995; Laasonen, Service, & Virsu, 2001). Consistent with this hypothesis, dyslexics have shown difficulties in perceiving a short gap between two stimuli, as in the case of a flicker (see, Au & Lovegrove, 2007; Slaghuis & Lovegrove, 1985) and in perceiving the displacement between visual frameworks, which is needed for movement perception (for difficulties with *motion coherence* see Hansen et al., 2001; for *motion transparency*, see Hill & Raymond, 2002; for *illusion of movement,* see Cestnick & Coltheart, 1999; but for negative findings also see Jones, Holly, Branigan, & Kelly, 2008). These difficulties, however, should affect only stimuli very close in time and space. To minimize the need for temporal resolution, our paradigm uses stimuli that are relatively widely separated in time or space.

## Spatial order and visual attention

As a group, dyslexics are impaired in tasks requiring visual attention (Iles, Walsh, & Richardson, 2000; Roach & Hogben, 2004), and difficulties with visual attention have been found to predict difficulty with literacy acquisition (Franceschini, Gori, Ruffino, Pedrolli, & Facoetti, 2012; Kevan & Pammer, 2009). Attention is clearly needed for reading (it must be moved along the words on the page as well as being distributed across the letters of a single word). The parietal lobes, which are involved in directing visual attention, may be damaged in dyslexia as the end point of a dorsal stream dominated by magnocellular inputs (Jones et al., 2008; Pammer & Vidyasagar, 2005; Vidyasagar & Pammer, 2009). Attention and encoding of order are not the same, but they are difficult to distinguish from one another. Two types of visual attention are important to encode order in visual arrays.

### Splitting attention

Dyslexics have difficulties in tasks involving processing of visual arrays (for arrays of consonants, see Bosse, Tainturier, & Valdois, 2007; Valdois, Bosse, & Tainturier, 2004; for arrays of digits, see Hawelka, Huber, & Wimmer, 2006; Hawelka & Wimmer, 2005; for arrays of nonalphanumeric characters see Jones et al., 2008; Pammer, Lavis, Hansen, & Cornelissen, 2004). We have confirmed these difficulties in a group of dyslexics largely overlapping with the ones studied here (Romani, Tsouknida, Di Betta, & Olson, 2011). We used a same–different task in which participants had to compare sequences of eight characters (letters or other alphanumeric symbols) presented next to one another and decide whether they were the same or different. Difficulties with spatial arrays could stem from impairments in encoding spatial order. However, like others, we have attributed these difficulties to reduced attention because performance was particularly poor for locations most susceptible to crowding and interference, where attention was most needed. Attention can normally be split into a number of spotlights to allow the encoding of information at several locations. If dyslexics have a reduced number of spotlights available, this will decrease their ability to encode order for crowded stimuli (for the hypothesis of a reduced attentional window see also Bosse et al., 2007; for evidence of crowding effects see Martelli, Di Filippo, Spinelli, & Zoccolotti, 2009; Moll & Jones, 2013; Perea, Panadero, Moret-Tatay, & Gómez, 2012; Zorzi et al., 2012). To minimize difficulties with crowding and splitting of attention, our experimental paradigm employs large, well-spaced, and distinct characters in the simultaneous condition.

### Shifting attention

Another type of attentional impairment that may explain difficulties in processing arrays is a difficulty in shifting attention from one position to the next. The so-called sluggish attentional shifting hypothesis of dyslexia or SAS (Hari & Renvall, 2001) combines deficits of visual attention with deficits of temporal resolution. A “prolonged attentional dwell time” will result in larger input chunks or time chunks being fed to the processing system, with a consequent loss of spatial or temporal resolution. The SAS hypothesis predicts deficits in processing visual arrays if attention cannot be disengaged from one stimulus and moved to the next. Supporting results come from different paradigms. Dyslexics have shown an extended “blind window” or *attentional blink*, which impairs processing of identical stimuli that are presented close to one another in a sequence (Buchholz & Aimola-Davies, 2007; Facoetti, Ruffino, Peru, Paganoni, & Chelazzi, 2008; Hari & Renvall, 2001; but also see Lacroix et al., 2005 for contrasting results). As another example, dyslexics have been found impaired when asked to count rapidly presented sequences of squares (Conlon, Sanders, & Zapart, 2004; Eden, Stein, Wood, & Wood, 1995). This poor performance is well explained by SAS. Clearly, stimuli cannot be counted effectively if attention cannot be allocated to each stimulus individually (see also Facoetti et al., 2008; Lallier et al., 2010, for supporting evidence and Lallier et al., 2009, for mixed results).

Studies that have reported results consistent with SAS have also presented stimuli very briefly (e.g., 10 stimuli/s in Hari & Renvall, 2001). With longer presentations, we found no evidence that adults were affected by SAS in an array matching task (Romani, Tsouknida, et al., 2011). Although they were very inaccurate in detecting differences in certain positions, they carried out the task with the same serial strategy and the same speed as the controls (reaction times [RTs] increased at the same rate across the positions of the array). Difficulties in disengaging attention, instead, predict a summing of delays across positions and increasing differences from controls. To minimize difficulties with SAS, we use relatively long stimulus presentations (200-ms presentation with 300 ms interstimulus interval, ISI, in the sequential condition). However, if SAS has an impact at all, it should equally affect the ordering and the recognition of characters presented sequentially.

## Abstract (long-term) order encoding

So far we have concentrated on how our paradigm will be able to distinguish deficits of temporal order and spatial order from alternative deficits. Here, we discuss the possibility of a third type of order-encoding skill, which involves a more permanent, long-term representation of order, and one that is more abstract. Lexical representations consist of combinations, in different orders, of a small set of units (phonemes or letters). Having properly specified lexical representations is important for reading and even more important for spelling, and difficulties with learning novel words are common in dyslexia (see for English children: Vellutino, Scanlon, & Spearing, 1995; for English adults: Di Betta & Romani, 2006; for German children: Mayringer & Wimmer, 2000; Wimmer, Mayringer, & Landerl, 1998; for Dutch-speaking children: Messbauer & de Jong, 2003). Lexical representations are likely to encode the order of subunits in an abstract way. This is because lexical representations are used for comprehension and production and are consolidated from stimuli presented in a variety of formats. Individuals with developmental dyslexia may suffer from a deficit in encoding *abstract order*, instead of, or in addition to, a deficit in encoding temporal or spatial order. This was explicitly hypothesized by Szmalec et al. (2011) who linked learning of serial order in a Hebb paradigm to learning of lexical representations (see also Page & Norris, 2009). Consistent with this hypothesis, we have shown that a task involving learning novel written words in association with pictures (*written lexical learning*) explains substantial variation in reading and spelling proficiency in a population of adults with dyslexia, independent of phonological skills (Di Betta & Romani, 2006; Romani et al., 2008; Romani & Stringer, 1994; Romani et al., 1999). Moreover, written lexical learning showed a striking asymmetry with phonological tasks in predicting orthographic skills. Written learning was most strongly associated with *word* reading and even more with word spelling, while phonological skills were mostly associated with *nonword* reading and spelling. These results suggest that dyslexics may suffer from a difficulty in encoding abstract order, which affects both the creation of new orthographic representations (written learning) and their retrieval (word spelling).^1^

1Note that learning mechanisms also interact with attentional and reward mechanisms, which may be impaired in dyslexia as outlined above (see Roelfsema, van Ooyen, & Watanabe, 2010).


In the present study, we want to examine the relation between abstract order learning skills and more peripheral, modality-dependent order-encoding mechanisms. The hypothesis that a single-order deficit underpins dyslexia predicts correlations between an order-reconstruction task and lexical learning and similar correlations between each of these tasks and orthographic tasks. The hypothesis of separate order-encoding skills still predicts correlations between task tapping order, but predict different patterns of associations with orthographic tasks, if contributions are different.

## Summary of predictions

Given what we have discussed, a specific deficit in *temporal order encoding* predicts:

1. Deficits in remembering order, even with visual, difficult-to-name characters (our H&J order-reconstruction task). In contrast, poor-quality phonological representations should only affect auditory stimuli or visual stimuli that are highly nameable.
2. Deficits in remembering order when the characters of a series are presented one after the other (sequential condition of the H&J task), but not when the same series is presented simultaneously. In contrast, poor encoding of *spatial order* predicts difficulties in the simultaneous condition of the task.
3. Deficits in remembering order, but not in recognition of individual characters (only deficits in the order-reconstruction component of the task). We minimize the contribution of poor temporal resolution or SAS to our task, but if these impairments have any impact at all, they should also affect recognition of sequentially presented characters.

Our experimental investigation assesses these predictions. In addition, it assesses the relative contribution of different tasks involving encoding of order to developmental dyslexia.

## Experimental Investigation

### Participants

The same participants were used throughout the study. Dyslexic participants (*N* = 44) were recruited mainly through posters affixed at Aston University, through student counselling centres at the University of Birmingham and Aston University, and through the Birmingham Adult Dyslexia Group. Control participants (*N* = 40) were recruited mainly though research participation schemes at both the University of Birmingham and Aston University. Older control participants were recruited though word of mouth; 3/40 were related to the dyslexic participants. A total of 27 dyslexics (61% of sample) were the same participants as those tested in Romani et al. (2008); the remaining were new participants. A subset of the participants involved in the present study also carried out the serial matching task described in Romani, Galluzzi, and Olson (2011). Among the dyslexics, 23 had a formal diagnosis of dyslexia, 13 were self-referred for suspected dyslexia, and eight started to be tested as controls, but were found to have significant impairments in reading and/or spelling tasks. These impairments mostly affected nonword processing, which could explain their clinical underdetection. Since our study investigates variation in both word and nonword processing, these individuals were included in the dyslexic group. They showed a cognitive profile similar to that of the other dyslexics, but were generally less severely impaired.

Participants were categorized as dyslexics if they had:

- Normal IQ on the Wechsler Adult Intelligence Scale–Revised (WAIS–R; Wechsler, 1981).
- Reading or spelling of either words or nonwords that was two standard deviations below the control mean. Since, it is important to consider possible speed–accuracy trade-offs, participants were considered impaired only if poor performance (≤2 *SD*s) in terms of speed was not compensated with above-average accuracy or vice versa.
- No history of psychological and/or neurological problems.

Testing was carried out in a quiet room at one of the participating universities. Each participant attended one to two weekly sessions, each lasting between one and two hours, over several months. An effort was made to test all participants with all tasks; a few data points, however, are missing for a few tasks.

## Dyslexic Classification and Performance in Reading and Spelling

### Method

#### Performance IQ (from the WAIS–R; Wechsler, 1981)

To obtain a measure of nonverbal cognitive skills, participants were asked to carry out all the nonverbal subtests of the WAIS–R. These included: picture completion (requiring detection of missing parts of familiar objects), picture arrangement (requiring the logical arrangement of a set of pictures depicting a story), block design (requiring the reproduction of abstract designs using cubes with white and red parts), object assembly (requiring assembly of a puzzle), and digit symbol transcoding (requiring translation of as many symbols as possible into numbers in a unit of time). Each subtest was administered, scored, and standardized according to the guidelines of the test, and a composite score (performance IQ) was computed for each participant.

#### Vocabulary and similarities subtests (from the WAIS–R; Wechsler, 1981)

As is commonplace in research on dyslexia, these tasks were also used as control tasks, tapping verbal lexical skills generally not impaired in dyslexia. In the Vocabulary subtest, participants were asked to explain the meaning of spoken words of increasing complexity (progressively less frequent and more abstract). In the Similarities subtest, they were asked to explain in what way two words could be regarded as similar.

#### Reading of text

Participants were asked to read aloud as fast and as accurately as possible a passage taken from a scholastic book (“How to prepare for SAT I” Brownstein, Weiner, & Weiner-Green, 1997). The passage was one and a half pages long and written in 12-point Times New Roman font with double-line spacing. An audio recording was made and was transcribed after the testing session. Mispronunciations or missing words or lines were noted by the experimenter. No feedback was provided. A similar passage was used to test reading comprehension. Participants were given 10 minutes to read a passage to themselves and answer nine multiple-choice questions (without referring back to the passage). Performance was measured by the number of correct responses.

#### Single-word and nonword reading

Three lists were used: List 1 included both real English words of various frequencies and nonwords (*N* = 80 each). The real words were taken from PALPA 31 (Kay, Lesser, & Coltheart, 1992). The nonwords were obtained by changing one or two letters in the corresponding words. List 2a, from Seidenberg, Waters, Barnes, and Tanenhaus (1984), Experiment 3, consisted of 52 words; List 2b, from Seidenberg et al. (1984), Experiment 4, consisted of 90 words. Both List 2a and List 2b included regular and irregular words of high and low frequencies. In total, 225 words and 80 nonwords were presented.

The words appeared one at a time at the centre of a Macintosh computer screen. They remained on the screen for 500 ms. Participants were asked to read them aloud as carefully and as quickly as possible and to say “don't know” only if they were unable to work them out. Words within each list were presented in a randomized order. Reaction times (RTs) were recorded via a voice-key. The experimenter wrote down each response. Misread words and “Don't know” responses were counted as errors. The RT results include only correct responses. Moreover, RTs more than 2 standard deviations from each participant's mean were considered outliers and were removed from the analysis.

#### Single-word and nonword spelling

We used word lists from: (a) Schonell (1985); (b) Holmes and Ng (1993); and (c) Romani and Ward (1995). These included regular and irregular words of various frequencies and lengths. There were 344 words in total. We also administered 24 monosyllabic nonwords from PALPA 45 (Kay et al., 1992). They were obtained by substituting one or two phonemes in real English words. The mean number of phonemes was 3.8 (*SD* = 0.6, range = 3–5). The pronunciation of the original word was used as guidance for that of the derived nonword.

A male native English speaker tape recorded all the words and nonwords (presented as blocked lists). The stimuli were presented one at a time with no time limit for the response. In case of self-corrections, only the last response was scored. Homophones were presented with a disambiguating sentence. Each misspelled word counted as one error. For nonwords, all phonologically plausible renditions of the items were accepted as correct (for instance, both BOKE and BOAK for BOAK, pronounced like “cloak”).

### Results

Results are reported in Table 1, which shows: (a) mean performance of the dyslexics and the control group, (b) *z* scores computed from the mean and the standard deviation of the control group, and (c) percentage of dyslexics impaired. The dyslexics did not differ from the controls in terms of age, education, performance IQ, and results in the Vocabulary and Similarities subtests of the WAIS–R. Instead, they differed significantly in all tasks involving reading text and reading and spelling single words and nonwords, consistent with a diagnosis of developmental dyslexia. Reading comprehension was less significantly impaired. This is consistent with an ability to compensate for word-decoding difficulties by capitalizing on good semantic and syntactic processing.

**Table 1.** Demographic and defining characteristics of the 44 dyslexics and 40 controls

Characteristics		Dyslexics				Controls		Comparison	
		Mean	*SD*	*z*-score	% impaired	Mean	*SD*	Value	*p*
Age	years	27.7	10.6	0.2	9.1	25.5	10.5	*F* = 0.9	*ns*
Sex	male:female	15:29	—	—	—	8:32	—	χ^2^ = 1.4	*ns*
Education	university:secondary	33:11	—	—	—	31:09	—	χ^2^ = 0.0	*ns*
Performance IQ	scaled score	107	12.1	0.1	0	108.8	13.8	*F* = 0.4	*ns*
Vocabulary	scaled score	10.6	2.3	0.1	4.5	10.7	2.4	*F* = 0.1	*ns*
Similarities	scaled score	12.8	2.7	−0.3	0	11.9	3	*F* = 2.1	*ns*
Spelling									
Words	% errors	23.3	11.7	4.8	77.2	8.1	3.1	*F* = 61.2	<.001
Nonwords	% errors	25.5	17	2.3	47.7	9.7	6.8	*F* = 30	<.001
Reading									
Words	RT	661	231	2.1	36.3	526	63	*F* = 12.7	.001
Words	% errors	5.9	3.4	3.3	65.9	2.2	1.1	*F* = 42.1	<.001
Nonwords	RT	1048	672	3.4	40.9	658	114	*F* = 13.1	<.001
Nonwords	% errors	27.8	13.2	5.6	86.4	7.2	3.7	*F* = 90.4	<.001
Reading text									
Speed	ms	243	100	1.8	29.5	179	36	*F* = 14.6	<.001
Accuracy (/466 words)	*N* errors	12.9	11.6	2.5	38.6	3.6	3.7	*F* = 23.2	<.001
Comprehetion (/9 questions)	*N* errors	4.7	2.1	0.6	11.4	3.6	1.9	*F* = 2.1	.01

*Note:* For text comprehension: 43 dyslexics and 38 controls; for text speed and accuracy: 40 dyslexics and 40 controls. Performance 2 standard deviations below the control mean is categorized as “impaired”. The sign of the *z*-scores for the scaled scores have been changed so that positive *z*-scores reflect worse performance matching the sign across errors and RT scores. RT = reaction time in ms.

As shown in Table 1, the great majority of our dyslexic participants were severely impaired in *word spelling* and *nonword reading accuracy*. Using a strict criterion to judge impairment (≤2 *SD*s from the control mean), four participants had normal word spelling, and five had normal nonword reading accuracy. More participants performed normally in nonword spelling and word reading accuracy and even more in word and nonword reading speed. Generally, however, performance was poor across reading and spelling. Using a more lenient criterion to judge impairment (≤1 *SD* from the control mean), only one participant performed normally in reading across the board, and one performed normally in spelling. For brevity, we refer to our reading/spelling-impaired group as dyslexics, since their profile is fully compatible with that of other groups of adult developmental dyslexics reported in the literature.

### Discussion

We used stringent criteria for inclusion in the dyslexic group (2 *SD*s below the mean). We based classification on alternative measures (reading and spelling of words and nonwords) and we wanted to be sure that the dyslexics were truly impaired. Our inclusion criteria, however, were more stringent than those used by other studies (see Hatcher, Snowling, & Griffiths, 2002; Swanson & Hsich, 2009), and, thus, it is possible that our dyslexic participants were more impaired than other adult dyslexic groups reported in the literature. Their profile of impairment, however, was not dissimilar.

As is typical, our dyslexic participants showed particularly severe difficulties with nonword reading (accuracy *z*-scores: nonwords = −5.6; words = −3.3; RT *z* scores: 3.4 and 2.1). These difficulties are commonly interpreted as arising from poor phonology and poor use of conversion rules (see Herrman, Matyas, & Pratt, 2006; Ijzendoorn & Bus, 1994). This interpretation, however, is not shared by everybody (see Facoetti et al., 2006; Landerl & Wimmer, 2000), and other aspects of our results argue against it. Poor conversion rules predict similar difficulties in nonword spelling, which, instead, was less impaired (accuracy *z-*scores: words = −4.8; nonwords = −2.3). This cannot be explained by this task being easier since performance was worse with nonword than with word spelling with both groups. Performance in nonword spelling was also very variable, however, and this makes it harder to demonstrate an impairment. Another piece of evidence against poor use of conversion rules, however, comes from the presence of a normal regularity effect in reading. We assessed a regularity effect by contrasting 86 regular words and 56 irregular words. There was no main effect of regularity with RTs, but a significant effect with accuracy in both groups (dyslexics: % error for regular words = 2.5; for irregular words = 12.5, *F* = 113, *p* < .001, *MSE* = 4.6; controls: % error for regular words = 0.6; for irregular words = 6.8, *F* = 100, *p* < .001, *MSE* = 2.1). Moreover, there was a significant interaction between group and regularity (*F* = 7.8, *p* = .006, *MSE* = 3.4) indicating that, in fact, the regularity effect was stronger in the dyslexics (see Metsala, Stanovich, & Brown, 1998; Mundy & Carroll, 2013, for consistent results indicating normal regularity effects in dyslexics).

Taken together, these results suggest that poor nonword reading in the dyslexics is not caused by poor conversion rules. A plausible alternative is a difficulty in encoding serial order (see Facoetti et al., 2010; Romani, Tsouknida, et al., 2011 for evidence consistent with this position). Encoding letter order is crucial for nonword reading, but less important for word reading where known, stored phonological representations can help “guess” the word on the basis of much more limited information. Poor encoding of order, in turn, may be caused either by a deficit of allocating attention, or by a primary deficit in encoding spatial order. Our experimental investigation is devoted to provide evidence for these different alternatives and their relation.

## Background Cognitive Profile

### Method

#### Phonological short-term memory (STM)

STM was investigated with three tasks that asked for repetition of sequences of stimuli (digits, words, or nonwords) in serial order. Stimuli were presented at a rate of about one per second. Digit Span lists ranged from four to eight digits (*N* = 10 sequences for each length). Testing at each length went on until the participant repeated fewer than three (out of 10) sequences correctly or until all sequences had been attempted. For scoring, a value of 0.1 was assigned to each sequence repeated correctly and added to a three point baseline. In word serial recall, the participant was read 10 series of five words. These were mono-, bi-, and trisyllabic words of medium–average frequency. In nonword serial recall, 30 triplets of nonwords that respected the phonotactic constraints of English were used. There were 10 sequences each of monosyllabic, bisyllabic, and trisyllabic nonwords. Performance was measured by the percentage of items recalled in the correct order.

#### Phonological awareness

Phonological awareness was investigated with two tasks commonly used in developmental dyslexia. The phoneme counting task (Perin, 1983) consisted of 48 stimuli: Thirty-two were real English words, and 16 were nonwords. The number of phonemes varied from two to five (four items for each length). The stimuli were spoken, one at a time, by the experimenter. Participants were asked to report the number of phonemes in each item with no time limit. The spoonerisms consisted of 70 pairs of real English words. Participants heard two spoken words and were asked to exchange the initial sounds to produce two different words (sock–rent → rock–sent), two nonwords (dare–night → nare–dight), or a word and a nonword (lost–dust → dost–lust). There was no time limit to respond. A point was awarded for each pair where both words were produced correctly.

#### Lexical learning

Participants had to learn the association between a made-up word and a picture of an object or animal (a black-and-white drawing). At the beginning of the learning phase, participants were presented with a number of pictures, each associated with a novel word (spoken or written in different blocked conditions). They were asked to repeat the word, if spoken, or to copy it down, if written. In the testing phase, they were asked to recall the correct novel word on presentation of the picture alone (to say it or to write it down, depending on task modality). Feedback was provided in case of errors. The task was discontinued when all the words in the lists were recalled correctly or after a maximum of five attempts at the whole list. Two lists were used in the spoken modality and two in the written modality. One included nonwords that respected English phono/orthotactics; the other included Dutch words, unfamiliar to all the participants. In the *written modality*, the list of nonwords consisted of nine stimuli (mean number of letters = 5.9, *SD* = 1.0; mean number of syllables 1.9, *SD* = 0.6). The list of Dutch words consisted of 24 stimuli (mean number of letters = 5.8, *SD* = 1.9; mean number of syllables = 1.7, *SD* = 0.8). In the *spoken modality*, the list of nonwords consisted of 10 stimuli (mean number of phonemes = 5.4; *SD* = 1.4; mean number of syllables = 2.0; *SD* = 0.7). The list of Dutch words consisted of 14 stimuli (mean number of phonemes = 5.1, *SD* = 2.0; mean number of syllables = 1.6, *SD* = 0.8). Performance was measured by the mean percentage of words produced correctly over five trials. When testing was discontinued after a completely correct list, all subsequent words were counted as correct.

#### The Visual Index of the Wechsler Memory Scale–Revised (WMS–R; Wechsler, 1987)

This combines results from three tasks. In the first task, participants are presented with matrices containing different combinations of rectangles of different sizes and shades of grey. They have to recognize them among close distractors (*n* = 10). In the second task, participants are asked to learn the associations between six colours and six nonsense shapes. The learning procedure is repeated three times. In the third task, participants are presented with four meaningless figures, one at a time, for 10 seconds. They have to draw each figure once it is removed from sight. Performance is measured by the number of features recalled.

#### The Doors and People Test—visual tasks (Baddeley, Emslie, & Nimmo-Smith, 1994)

The visual learning test is a test of visuospatial memory. Participants are asked to copy a series of four designs and then to draw them from memory after a filled delay. The visual recognition test involves viewing two series of photographs of doors (*N* = 12 each). Then, participants have to recognize each target door among a group of four very similar doors. What kinds of memory resources are necessary for this subtest is less clear, but it contrasts with other visual tasks in that good performance depends on veridical memory for details.

### Results and discussion

Results are presented in Tables 2 and 3. As expected, our dyslexics were impaired on a variety of tasks tapping the processing and retention of phonological representations. These impairments were generally of medium severity with the exception of performance on the Spoonerisms task, where the impairment was very severe. This could be due to the fact that this task relies not only on phonological representations but also on orthographic representations—which are impaired in dyslexics (see Castles & Coltheart, 2004). The dyslexics were also impaired in tasks of lexical learning, consistent with previous results (Di Betta & Romani, 2006; Romani et al., 2008; Romani & Stringer, 1994; Romani et al., 1999). As expected, performance was completely normal in the tasks tapping visual memory (see also Hawelka & Wimmer, 2005; Shovman & Ahissar, 2006; Von Karolyi et al., 2003).

**Table 2.** Performance of the group of the 44 dyslexics and 40 matched controls on phonological processing and lexical learning tasks

Tasks		Dyslexics				Controls		Comparison	
		Mean	*SD*	*z* score	% impaired	Mean	*SD*	*F*(1, 69–70)	*p*
Phonological STM									
Digit span	Raw score	5.7	0.8	1.5	30	6.8	0.7	38.9	<.001
Nonword serial recall	% errors	34.1	14.1	1	23	23.9	10.5	13.8	<.001
Word serial recall	% errors	37.8	10.8	1	18	27.9	10.1	19	<.001
Phonol. awareness									
Phoneme counting	% errors	18.8	16.4	0.9	23	9.8	10.3	9	.004
Spoonerisms	% errors	22.0	20.2	3.0	54	5.4	5.6	25.2	<.001
Lexical learning									
Spoken	% errors	65.3	14.7	1.5	36	44.8	13.8	43.4	<.001
Written	% errors	52.3	19.7	1.6	39	27.9	15.0	39.9	<.001

*Note:* Performance 2 standard deviations below the control mean is categorized as “impaired”. The sign of the *z*-scores for the digit span has been changed so a positive *z*-score reflects worse performance, as do percentages of errors in other tasks. STM = short-term memory; Phonol. = Phonological.

**Table 3.** Performance of the dyslexics and matched controls on visuospatial memory tasks

Tasks		Dyslexics					Controls			Comparison	
		Mean	*SD*	*N*	*z*-score	% impaired	Mean	*SD*	*N*	*F*(1, 75–80)	*p*
WMS–R											
Visual Memory	index score	60.7	5	42	0.4	4.5	63.0	5.5	39	3.9	*ns*
Visual D&P											
Learning–immediate	% errors	1.9	5.4	39	−0.2	2.3	3.6	7	37	1.4	*ns*
Learning–delayed	% errors	4.1	8.1	39	0.0	2.3	4.5	8.5	37	0.1	*ns*
Recognition	% errors	22.4	15.1	39	0.4	9.1	17.8	12	37	2.2	*ns*
H&J											
Recognition	% errors	25.1	5.6	44	0.0	2.3	24.8	6.5	40	0	*ns*

*Note:* Performance 2 standard deviations below the control mean is categorized as “impaired”. The sign of the *z*-scores for the Visual Index has been changed so that a positive *z*-score reflects worse performance, as do percentages of errors in other tasks. WMS–R = Wechsler Memory Scale–Revised; D&P = Doors and People Test; H&J = Hindi and Japanese.

**Figure 1.**
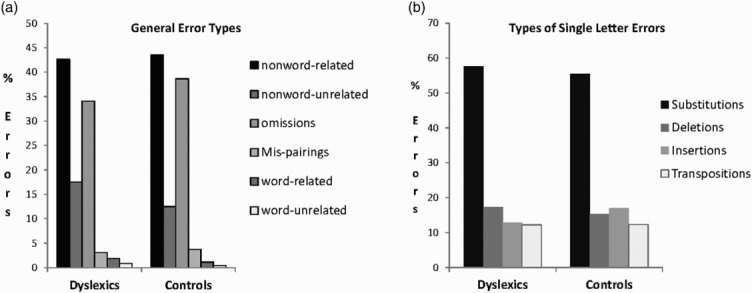
Percentages of different types of errors (over total errors) made in the written learning paired-associate task by dyslexic and control participants. (a) General error types. (b) Types of single letter errors.

Results for the written learning task were also analysed for type of error. In a first analysis, we distinguished errors according to whether they produced an existing word (phonologically related or unrelated to the target), produced a different nonword, or were mispairings where one of the stimuli to be learned was produced in response to the wrong picture. In a second analysis, we analysed single letter errors in terms of proportions of substitutions, deletions, insertions, and transpositions. Results are presented in Figure 1. Although dyslexic participants made many more errors, error patterns were very similar in the two groups.

Our experimental investigation is subdivided in two parts. In the first part, we assess the ability to recall the order of visual series of stimuli and recognize individual characters; here we contrast a temporal–sequential condition with a spatial–simultaneous condition. In the second part, we assess the interrelation between these tasks and tasks of orthographic processing, phonological processing, and lexical learning.

## Encoding Serial Order

### Method

To assess the ability to encode the order of a series of visual stimuli, we used an order-reconstruction task very similar to that originally employed in the single-case study of A.W. (Romani et al., 1999). The task involved the order reconstruction of either four Hindi characters or five Japanese characters, unfamiliar to the participants. The Hindi series were presented first; the Japanese series were presented in a separate session, a few days later. Each session lasted 25–30 min. Stimuli were presented on the computer, and participants were seated about 60 cm from the screen. Presentation of each sequence was preceded by a fixation cross that remained on the screen for 200 ms.

The series of four Hindi characters were drawn from a set of 40 characters, and the strings of five Japanese characters were drawn from a set of 50 characters.^2^ Characters were never repeated within conditions (simultaneous or sequential, see later), but the same characters were used in different combinations across conditions to make them comparable. Immediately after presentation of each series, participants were given a set of tiles with the characters just presented arranged in a random order, and they were asked to rearrange them to reproduce the original series. There were no time constraints to produce an answer. Everybody, however, rearranged the tiles quite quickly.

2We used Hindi and Japanese characters to assess generalization across type of stimuli. We used longer sequences with Japanese characters since they appeared easier to discriminate and to remember when we piloted the test.


With both Hindi and Japanese stimuli, simultaneous and sequential presentation conditions were contrasted. In the simultaneous condition, characters were presented all together in a single line at the centre of a computer screen. In the sequential condition, the characters appeared on the centre of the screen, one at time; presentation of each new character replaced the older one. For each condition and type of stimuli, 10 series of characters were presented. The order of the simultaneous and sequential conditions was counterbalanced across blocks of five series.

We equated overall exposure to the characters in the two conditions as much as possible. In the simultaneous condition, the four Hindi characters remained on the screen for 1700 ms. In the sequential condition, each character was presented for 200 ms with a 300 ms, unfilled ISI. Since there was no masking, it is reasonable to assume that processing continued during this period: thus, (200 × 4) + (300 × 3) = 1700. The five Japanese characters remained on the screen for 2200 ms in the simultaneous condition. Again, each character in the sequential condition remained on the screen for 200 ms with a 300 ms ISI: thus, (200 × 5) + (300 × 4) = 2200.

In the simultaneous condition, the four Hindi characters subtended 11.42° of visual angle (12 cm viewed at approximately 60 cm distance), the five Japanese characters subtended 15.19° of visual angle (16 cm). Each character was separated from the next by a blank space corresponding to 1.91° of visual angle (2 cm). In the sequential condition, each character subtended 1.43° of visual angle for height and 1.43° of visual angle for width (about 2 cm each).

The first block of each condition was preceded by a practice trial. Each character recalled in the correct order received one point in the scoring. A sample of the stimuli is presented in the Appendix. After each block, a recognition task was carried out involving the characters just presented (20 Hindi characters or 25 Japanese characters) intermixed with an equal number of new, distractor characters. The characters appeared on the computer screen one at a time, and participants had to press “Yes” for familiar and “No” for unfamiliar stimuli. Each character disappeared as soon as a response was made. Participants were asked to perform the task as accurately as possible with no time constraints.

### Results

Results are presented in Table 4. The pattern across the Hindi and Japanese versions of the task was very similar. In the order-reconstruction tasks, the dyslexics performed normally in the simultaneous condition, but significantly worse than the matched controls in the sequential condition. In the recognition tasks, involving recognition of the same individual characters as those used in the order reconstruction tasks, the dyslexics performed normally, both when the characters were previously presented together in a row (simultaneous condition) and when they were presented one at a time (sequential condition).

**Table 4.** Performance of the 44 dyslexics and 40 matched controls on the order-reconstruction and recognition conditions of the Hindi and Japanese tasks

Tasks	Dyslexics				Controls		Comparison	
	Mean	*SD*	*z*-score	% impaired	Mean	*SD*	*F*(1, 83)	*p*
Order reconstruction								
Simultaneous								
Hindi	34.4	13.6	0.2	5	32.0	11.8	0.7	*ns*
Japanese	45.5	10.3	0.2	0	42.4	15	1.2	*ns*
Total	40.5	9.4	0.2	0	37.8	11.5	1.5	*ns*
Sequential								
Hindi	28.1	14.2	0.9	14	16	14.1	15.4	<.001
Japanese	28.5	14.1	0.8	18	18	13	12.7	.001
Total	28.4	12.7	0.9	16	17.1	12.2	17.2	<.001
H&J recognition								
Simultaneous								
Hindi	28.5	6.7	0.1	2	27.5	8.1	0.4	*ns*
Japanese	26.9	7.1	−0.2	0	28.6	7	1.2	*ns*
Total	27.6	6	−0.1	0	28.1	6.9	0.1	*ns*
Sequential								
Hindi	24.6	6.3	0.3	2	22.5	7.7	2.0	*ns*
Japanese	20.8	7.5	0	2	20.7	7.5	0.0	*ns*
Total	23.6	6	0.1	0	21.5	6.7	0.6	*ns*

*Note:* Results are all in percentage of errors. H&J = Hindi and Japanese.

Averaging rates of correct responses between the Hindi and Japanese versions, we carried out two mixed analyses of variance (ANOVAs): one for the order-reconstruction task and one for the recognition task. In each ANOVA, condition was a within-subjects variable (simultaneous vs. sequential), and group was a between-subjects variable (dyslexics vs. controls). The order-reconstruction task showed a significant effect of condition (with the sequential condition being easier), *F*(1, 82) = 238.5, *p* < .001, *MSE* = 47.3, a significant effect of group (with the dyslexics performing worse), *F*(1, 82) = 9.5, *p* = .003, *MSE* = 215.9, and a significant interaction between condition and group, *F*(1, 82) = 15.9, *p* < .001. As shown in Table 4, the dyslexics performed worse than the controls in the sequential condition, but not in the simultaneous condition. Since the sequential condition was easier for both groups [dyslexics: *F*(1, 43) = 58.9, *p* < .001, *MSE* = 55.3; controls: *F*(1, 39) = 221.5, *p* < .001, *MSE* = 38.5], this interaction cannot be attributed to a difference in difficulty. Equally, the simultaneous condition is well off ceiling in both groups, and, thus, the generally good performance in the dyslexics cannot be accounted for by this condition being either too difficult or too easy.

The recognition task showed a significant effect of condition with the sequential condition again being easier, *F*(1, 82) = 184.0, *p* < .001, *MSE* = 7.9, but no effect of group, *F*(1, 82) = 0.04, *p* = .84, *MSE* = 73.5, and no significant interaction between condition and group, *F*(1, 82) = 3.0, *p* = .08. As shown in Table 4, the dyslexics performed as well as the controls in both the sequential and the simultaneous conditions.

**Figure 2.**
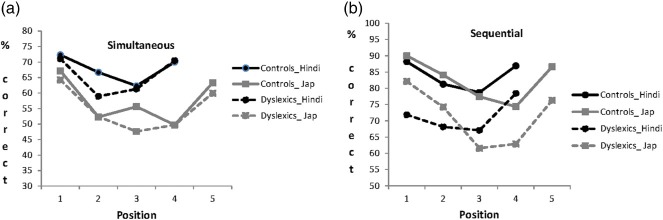
Serial position curves in the Hindi and Japanese order reconstruction task by condition and group of participants. (a) Simultaneous condition. (b) Sequential condition.

The serial position curves for the two conditions of the order-reconstruction task are shown in Figure 2. In both conditions and for both groups, the serial position curves are roughly U-shaped with better performance for the initial and final positions and worse performance for the intermediate positions. In the sequential condition, there were numerical differences across all positions although significance was reached only for Position 1 with four-character arrays (χ^2^ = 4.1, *p* = .04) and Position 3 with five-character arrays (χ^2^ = 4.3, *p* = .04). In the simultaneous condition, the two groups performed similarly across positions except for Position 2 with four-character arrays and Position 3 for the five-character arrays. This last difference reached statistical significance (χ^2^ = 4.1, *p* = .04). Control participants showed an advantage for the central location not shown by the dyslexic participants.

### Discussion

These results replicated what we observed in the single case study of A.W. (Romani et al., 1999). In the order-reconstruction task, there was a significant impairment in the sequential condition, but no impairment in the simultaneous condition, consistent with a deficit in processing temporal order. The finding of no overall deficit in the simultaneous condition is consistent with other studies that have shown that dyslexics have no overarching difficulties with visuospatial processing, but perform poorly only in conditions that stress attention by requiring split allocation to crowded positions (see Romani, Tsouknida, et al., 2011).

In our adult group, only about a third of dyslexic participants were impaired in the sequential order task (34% dyslexics performed more than 1.5 *SD*s below the control mean, and 16% showed severe impairments with performance more than 2 *SD*s below the control mean). This proportion is smaller than that returned by phonological tasks and lexical learning. One can note, however, that all tasks (including phonological tasks) produced impairments only in a subset of participants. Moreover, most of the tasks identified by the literature to be problematic for dyslexics involve phonological and/or orthographic representations that are directly involved in reading and spelling. The order reconstruction task involved neither, and, thus, impairments in this task are especially significant. The question of the overlap of deficits is addressed in the remaining sections of the paper.

We attribute the worse performance in the sequential condition to a deficit of temporal order. Alternative hypotheses are unlikely, as outlined below. *Slow visual processing* (e.g., Breznitz & Meyler, 2003; Keen & Lovegrove, 2000), *poor visual memory* (Ram-Tsur, Faust, & Zivotofsky, 2008), *reduced temporal resolution* (e.g., Laasonen et al., 2001), *sluggish attentional shifting* (e.g., Hari & Renvall, 2001), and *deficits of temporal resolution* (Stein, 2003) all predict deficits in tasks involving visuospatial processing and/or recognition of individually presented characters, contrary to what we found. A *reduced attentional window*, as suggested by Valdois et al. (2004), predicts worse performance in the simultaneous than in the sequential condition, also contrary to what we found.

Note that we specifically tested the hypothesis of a deficit in visual processing speed in Romani, Tsouknida, et al. (2011) with a subset of the dyslexics tested here and found no impairment. We used a matching task where participants had to decide whether two strings of eight letters or symbols (e.g., %, &, £, etc.) presented next to one another on the computer were the same or different. The comparison between the two strings was carried out with some seriality as demonstrated by RTs progressively increasing with position of the difference along the string. However, even in the conditions where dyslexics were slightly worse than the controls, differences did not increase across the string. This showed that dyslexics processed characters at the same speed as controls, otherwise differences would have summed across positions and become increasingly larger. Also, note that with our paradigm we have purposely tried to minimize the impact of these deficits. Our results are consistent with a nonsignificant impact. The results of Lassus-Sangosse, N'guyen-Morel, and Valdois (2008) nicely complement our own by showing that dyslexics performed normally when they were asked to recall letter sequences *disregarding order*.

Our results are also difficult to account for in terms of general attentional deficits or naming difficulties. *Lapses of attention* could impact the sequential condition more because here rescanning is not a possibility. Lapses of attention, however, predict other characteristics of performance that we did not find, such as larger standard deviation in the sequential condition and errors homogeneously distributed across positions (see also Davis, Castles, McAnally, & Gray, 2001, for evidence against this hypothesis). Finally, Hawelka and Wimmer (2008) have argued that difficulties in processing visual arrays may be explained with difficulties in *verbal coding*.^3^ Naming the characters could also be helpful in our task, allowing the use of verbal short-term memory, and the sequential condition may make it easier to name the characters and use verbal memory. The dyslexics, therefore, could have performed worse in this condition because of their *poorer verbal working memory*. This explanation is unlikely for two reasons. We chose characters that would be difficult to name, the set was large, and resampling was minimal, with each character only used twice and the order tasks always preceding the recognition task (so that all characters were new at this point), all of which should have reduced the utility of a naming strategy. More crucially, however, if the dyslexics had more difficulties in using a naming strategy and short-term memory in the sequential condition, they should have also performed poorly when they were asked to recognize the characters. In the case of recognition, instead, they performed as well as the controls, showing, like them, an advantage for sequential presentation (see also Holmes, 2006; Holmes et al., 2008, for evidence that a naming strategy is little used with this task). Therefore, difficulties with naming and working memory cannot explain the selective difficulty shown by dyslexics with the sequential order-reconstruction condition.

3They have reported that a group of adult German dyslexics performed normally when arrays of pseudoletters were used as stimuli, instead of arrays of digits, as in their original paradigm. However, their nonverbal task required only the identification of a single character embedded in a row of other characters. Thus, normal performance indicates normal visual memory in dyslexia, consistent with our own results, but does not address the hypothesis of a deficit in encoding temporal order.


## Relation among Tasks

The hypothesis that a *single* deficit of order encoding is the cause of developmental dyslexia predicts that different tasks tapping order encoding should be associated with one another, but also be similarly associated with reading and spelling tasks. Instead, if developmental dyslexia is caused by a *family* of deficits all having to do with order encoding but affecting orthographic processing in different ways, then pattern of correlations may be different for different type of tasks related to order encoding. *Spatial* order encoding should be more important for reading and *temporal* order encoding for spelling. Moreover, order encoding may be particularly important for spelling. Converting a phonological representation into letters, as is done in spelling, is more time consuming than the reverse process in reading, and articulatory constraints, which play an important role in keeping phonemes in order in speaking, are not available in spelling. Consistent with these considerations, *errors of order* are much more common in written than in spoken word production (see Romani, Galluzzi, & Olson, 2011 for results with aphasic patients). Finally, nonword spelling may be even more dependent on temporal order encoding. Nonpractised novel sequences may be converted through smaller chunks so that keeping track of order (i.e., keeping track of which units are already converted and which still need conversion) is more taxing.

### Method

To simplify our variables, we extracted a single phonological factor from a factor analysis that included digit span, nonword serial recall, phoneme counting, and the spoonerisms. A single factor with an eigenvalue greater than 1 was returned, which accounted for 68.9% of variance and had high loadings on all the components: digit span, .85; nonword serial recall, .89; phoneme counting, .79; and spoonerisms, .79. We also derived a single visuospatial factor using the Doors and People Immediate Learning, the Doors and People Delayed Learning, and the Visual Index of the Wechsler Memory Scale. The recognition subtests of the Doors and People Test were not included since they do not tap visuospatial memory to the same extent (see earlier). A single factor with an eigenvalue greater than 1 was returned, which accounted for 60.0% of variance and had high loadings on all the components: Doors and People Immediate Learning, .74; Doors and People Delayed Learning, .79; and the Visual Index, .79. We expect the visuospatial factor to correlate with both the sequential and the simultaneous condition since they both require memory for visual shapes. We report results with written learning only since results with spoken learning are similar, but provide a weaker contrast with the phonological factor.

### Results

#### Correlations

Correlations between the two order-reconstruction tasks, phonological factor, written learning, and orthographic tasks are presented in Table 5. Age has been partialled out. To correct for multiple tests across eight different orthographic tasks we used the Holm–Bonferroni correction (Holm, 1979). Similarly, we corrected for multiple tests across our six predictor tasks.

**Table 5.** Pearson two-tailed correlations partialling out age

	Whole group							
Tasks	H&J simul.	*p*	H&J sequ.	*p*	Phon. factor	*p*	Wr. lex. learning	*p*
Spelling								
Words	**.** **28**	.01	**.** **42**	<.001	**.** **56**	<.001	**.** **67**	<.001
Nonwords	**.** **28**	.01	**.** **50**	<.001	**.** **65**	<.001	**.** **45**	<.001
Reading								
Words RT	**.** **23**	.04	**.** **31**	.005	**.** **53**	<.001	**.** **41**	<.001
Word errors	**.** **24**	.03	**.** **34**	.002	**.** **45**	<.001	**.** **65**	<.001
Nonword RT	.17	.13	**.** **27**	.01	**.** **59**	<.001	**.** **34**	.001
Nonword errors	**.** **27**	.02	**.** **35**	.001	**.** **65**	<.001	**.** **62**	<.001
Text speed	.07	.53	.20	.08	**.** **43**	.002	**.** **42**	<.001
Text errors	.17	.14	**.** **30**	.008	**.** **56**	<.001	**.** **53**	<.001
H&J order reconst.								
Simultaneous	1.00	—						
Sequential	**.** **61**	<.001	1.00	—				
Phonological factor	**.** **41**	<.001	**.** **45**	<.001	1.00	—		
Wr. lex. learning	**.** **32**	.003	**.** **50**	<.001	**.** **43**	<.001	1.00	—
Visuospatial tasks								
H&J recognition	**.** **49**	<.001	**.** **51**	<.001	.17	.13	**.** **28**	.01
Visuospatial factor	**.** **43**	<.001	**.** **38**	.001	.22	.06	**.** **32**	.005
	*Dyslexic group*							
*Tasks*	*H&J simul*.	p	*H&J sequ*.	p	*Phon factor*	p	*Wr. lex. learning*	p
Spelling								
Words	.32	.04	.25	.11	.34	.02	**.** **55**	<.001
Nonwords	.24	.11	.33	.03	**.** **56**	<.001	.07	.69
Reading								
Word RTs	.14	.36	.11	.48	**.** **43**	.004	.26	.09
Word errors	.25	.11	.12	.42	.17	.28	**.** **56**	<.001
Nonword RTs	.20	.25	.13	.41	**.** **56**	<.001	.17	.28
Nonword errors	**.** **42**	.005	.09	.57	**.** **47**	.001	**.** **40**	.008
Text speed	.25	.12	.17	.29	.36	.02	.31	.06
Text errors	.13	.45	.11	.51	.34	.03	**.** **43**	.006
H&J order reconst.								
Simultaneous	1.00	—						
Sequential	**.** **53**	<.001	1.00	—				
Phonological factor	**.** **48**	.001	**.** **31**	.04	1.00	—		
Wr. lex. learning	.34	.03	**.** **39**	.009	.12	.49	1.00	—
Visuospatial tasks								
H&J recognition	.37	.06	**.** **51**	.002	.10	.51	**.** **39**	.009
Visuospatial factor	.28	.10	**.** **48**	.004	.27	.11	**.** **48**	.004
	*Control group*							
*Tasks*	*H&J simul*.	p	*H&J sequ*.	p	*Phon. factor*	p	*Wr. lex learning*	p
Spelling								
Words	**.** **38**	.01	.11	.49	.33	.04	.28	.08
Nonwords	**.** **43**	.007	**.** **53**	.001	.25	.12	.27	.09
Reading								
Word RTs	**.** **55**	<.001	**.** **47**	.008	.36	.02	.17	.28
Word errors	.27	.09	.11	.49	.33	.04	.16	.33
Nonword RTs	.24	.14	.23	.15	.24	.13	.14	.39
Nonword errors	.00	.97	.01	.92	**.** **46**	.003	.19	.25
Text speed	.23	.19	.12	.50	−0.2	.90	.07	.67
Text errors	.06	.73	.00	.96	**.** **74**	<.001	.10	.53
H&J order reconst.								
Simultaneous	1.00	—						
Sequential	**.** **71**	<.001	1.00	—				
Phonological factor	**.** **36**	.02	.29	.07	1.00	—		
Wr. lex. learning	**.** **31**	.05	.29	.07	.30	.07	1.00	—
Visuospatial tasks								
H&J recognition	**.** **58**	<.001	**.** **59**	<.001	.33	.04	.27	.09
Visuospatial factor	**.** **53**	.001	**.** **48**	.009	.32	.06	**.** **38**	.02

*Note:* For whole group, *df* = 84–70; for the dyslexic group, *df* = 37–41; for the control group, *df* = 34–37. Correlations which remain significant after the Holm-Bonferroni correction are in bold. These corrections are staged; with 8 comparisons the most significant correlation needs to have a *p* < .006, with 6 comparisons a *p* < .008. Phon. = phonological; simul. = simultaneous; sequ. = sequential; reconst. = reconstruction; RT = reaction time; wr. lex. = written lexical; H&J = Hindi and Japanese.

Results are complex, but general patterns are clear. When the whole group was considered together, as expected, there were extensive correlations between both the phonological factor and written learning, on one side, and orthographic tasks on the other. Crucially, there were also extensive correlations between the *sequential* H&J task and most orthographic tasks. Correlations with spelling and nonword spelling were particularly high. Finally, there were also high intracorrelations between the order-reconstruction tasks, the phonological factor, and written learning, consistent with the hypothesis of overlap in the tasks involving order.

When the two groups were considered separately, the number of significant correlations decreased, as one would expect given reduced variability and more noise in the data. This, however, revealed some specific patterns*.* In the dyslexics, both the phonological factor and written learning continued to show extensive correlations with orthographic tasks. The simultaneous H&J task correlated significantly with nonword reading, consistent with it tapping spatial order and allocation of attention; the sequential H&J task just missed significance with nonword spelling (after Bonferroni correction), consistent with it tapping encoding of temporal order. In the controls, the phonological factor remained associated with orthographic tasks (especially nonword reading and text reading), but, in striking contrast, written learning showed no association. Instead, both of the order-reconstruction tasks showed some strong associations with orthographic tasks, particularly nonword spelling and word reading speed. Across groups, there were correlations between the order-reconstruction tasks and tasks of visual memory, as expected.

If we compare the size of the correlations across groups, *written learning* makes a larger contribution to orthographic tasks in the dyslexics (Pearson *R* with: *word spelling*, dyslexics = .55, controls = .28, *p* = .14; *word reading accuracy*, dyslexics = .56, controls = .16, *p* = .04) while the *sequential Hindi and Japanese task* makes a larger contribution in the controls (Pearson *R* with: *word spelling*, dyslexics = .33, controls = .53, *p* = .14; *word reading speed*, dyslexics = .11, controls = .47, *p* = .08; all comparisons using Fisher *r* to *z* transformations). Although individually these differences may fail to reach significance, they reinforce each other in indicating that the pattern of correlations differs in dyslexics and controls.

#### Regression results

We ran stepwise regressions where we entered age in the first step and, at the second step, written learning, the phonological factor, and either the sequential or the simultaneous H&J task.

In the *whole group*, as expected, written learning was the best predictor of word spelling (*R*^2^ = .43, *p* < .001) and word reading accuracy (*R*^2^ = .34, *p* ≤ .001), which are tasks with strong lexical components. The phonological factor was the best predictor of nonword spelling (*R*^2^ = .41, *p* < .001), nonword reading speed and accuracy (*R*^2^ = .31 and *R*^2^ = .42, *p* ≤ .001), word reading speed (*R*^2^ = .24, *p* ≤ .001), and text reading speed and accuracy (*R*^2^ = .16 and *R*^2^ = .29, *p* < .001). The sequential H&J task made some independent contribution to nonword spelling (*R*^2^ = .04, *p* = .03).

In the *dyslexic group*, written learning was the best predictor of word spelling (*R*^2^ = .27, *p* < .001), word reading accuracy (*R*^2^ = .26, *p* < .001), and text reading accuracy (*R*^2^ = .18, *p* = .006). The phonological factor was the best predictor of nonword spelling (*R*^2^ = .36, *p* < .001), nonword reading speed (*R*^2^ = .26, *p* < .001), nonword reading accuracy (*R*^2^ = .24, *p* < .001), word reading speed (*R*^2^ = .15, *p* = .004), and text reading speed (*R*^2^ = .10, *p* = .03). The order reconstruction tasks made no independent contribution.

In the *control group*, the phonological factor was the best predictor of word reading accuracy (*R*^2^ = .08; *p* = .04), nonword reading accuracy (*R*^2^ = .13, *p* = .02), and text reading accuracy (*R*^2^ = .45, *p* < .001). In striking contrast, written learning made no contribution. Crucially, order tasks made a number of significant contributions. The simultaneous H&J task was the best predictor of word spelling (*R*^2^ = .12; *p* = .03) and word reading speed (*R*^2^ = .30, *p* < .001). The sequential H&J task was the best predictor of nonword spelling (*R*^2^ = .21, *p* < .001).

### Summary and discussion

Our correlation and regression analyses show three main results: (a) Tasks tapping different aspects of order encoding are strongly intercorrelated. (2) Tasks tapping different aspects of order encoding are associated with orthographic tasks. In addition, however, (c) patterns of associations differ by type of orthographic task and by group. Overall, these results point to the importance of considering not only whether correlations are present or absent, but also how they are modulated depending on the orthographic tasks and the participant group. The fact that correlation patterns differ for different skills related to order encoding suggests that developmental dyslexia is caused by a family of independent skills rather than a by a single processing deficit (see also Pennington, 2006; Peterson, Pennington, & Olson, 2013). The different patterns associated with the different tasks are outlined below.

*Phonological tasks* tap mainly the *quality of the acoustic/phonological representations,* which may help with retaining order in verbal tasks, but is not an ordering mechanism per se. Good-quality phonological representations are essential to guarantee a link with the corresponding orthographic representations and vice versa. Consistent with this hypothesis, correlations between a phonological factor and orthographic skills were extensive in the dyslexics and in the controls. In addition, however, correlations with nonword processing were particularly strong in the dyslexics, consistent with the importance of sublexical phonology and short-term memory for these tasks.

*Lexical learning* taps mainly *long-term, abstract encoding of order,* important for lexical consolidation. This skill is important for storing accurate, detailed orthographic representations, which are particularly important for word spelling. The strong selective association between written learning and accuracy in spelling and reading of words is consistent with this hypothesis (see also Di Betta & Romani, 2006; Romani et al., 2008). In the whole group, lexical learning was strongly correlated with the sequential order-reconstruction task, consistent with the hypothesis that these two tasks tap a common order-encoding component, as hypothesized by Szmalec et al. (2011), as well as independent skills.

The *sequential reconstruction task* is mainly associated with *temporal order encoding*. This explains the selective association shown with nonword spelling. Nonword spelling requires that a phonological representation be held in working memory, but also that this representation is constantly updated in relation to the evolving written representation (one has to keep track of which part of the representation has already been converted into letters and which is the “current” part that needs conversion). Temporal order encoding is crucial to this updating.

*The simultaneous reconstruction task* correlated with orthographic tasks across groups (with spelling in both groups, with word reading in the controls, and with nonword reading in the dyslexics). This task taps *encoding of spatial order*, but also allocation of *visuospatial attention*, and disentangling these skills is difficult. However, the fact that dyslexics perform well on this task, except for central locations, which are the most susceptible to crowding, points to an attentional component. This well explains the correlation with nonword reading, which requires fine deployment of attention to individual letters.

Correlation patterns differ not only by task, but also across participant groups. Lexical learning predicts orthographic proficiency only in the dyslexics. This suggests that the capacity to store orthographic patterns in long-term memory (lexical learning) may be especially important to compensate for more peripheral attentional and order-encoding difficulties. Good learning will guarantee that whatever is encoded is not lost and that information will accumulate over learning episodes. Instead, even good peripheral skills do not guarantee good long-term storage, which is crucial for accurate spelling and fast reading. The controls have good learning skills (they are mostly university students), and, thus, in this group, orthographic proficiency is more related to peripheral encoding skills. It is also important to note that correlations may be found not only with skills that are impaired but also with skills that are preserved. The dyslexics have good visuospatial memory, and they can use this to compensate for other deficiencies in orthographic tasks. Thus, the visuospatial factor correlated with word and nonword reading in the dyslexics (*r* = .32, *p* = .06, and *r* = .46, *p* = .006, respectively), but not in the controls (word reading accuracy, *r* = .14, *p* = .41; nonword reading accuracy, *r* = .08, *p* = .62). These results are important. They show that relations between tasks are not fixed. How strongly a skill correlates with a task depends not only on how useful it normally is for that task, but also on whether it continues to be used in the face of impairment and/or on whether it has an additional role in compensating for other impaired abilities.

## Predicting Group Classification

If written learning and the phonological factor tap different components, using them together should improve discrimination of controls from dyslexics. Instead, if the sequential H&J and written learning overlap in tapping an order component, using them together should not improve discrimination. To test this hypothesis, we used a number of binary regression analyses with group as the dependent variable and written learning, the phonological factor, and the H&J sequential task as the predicting variables. Results are reported in Table 6.

**Table 6.** Results of binary logistic regressions predicting the classification of our participants to the dyslexic and control groups

		Predicted											
		Written learning				Phonological factor				H&J sequential			
		*N* 1	*N* 2	Total %	*p*	*N* 1	*N* 2	Total %	*p*	*N* 1	*N* 2	Total %	*p*
Observed	Controls 1	**29**	11	72.5		**29**	11	72.5		**28**	12	70.0	
Observed	Dyslexics 2	10	**34**	77.3		13	**31**	70.5		16	**28**	63.6	
	Total			**75**.**0**				**71**.**4**				**66**.**7**	
	Wald			2.5	<.001			17.4	<.001			12.6	<.001
		*Written learning + phonological factor*				*Phonological factor + H&J sequential*				*Written learning + H&J sequential*			
		N *1*	N *2*	*Total %*	p	N *1*	N *2*	*Total %*	p	N *1*	N *2*	*Total %*	p
Observed	Controls 1	**33**	7	82.5		**29**	11	72.5		**28**	12	70	
Observed	Dyslexics 2	7	**37**	84.1		9	**35**	79.5		10	**34**	77.3	
	Total			**83**.**3**				**76**.**2**				**73**.**8**	
	Wald writt. learning			11.2	.001			—	—			13.8	<.001
	Wald phon. factor			8.4	.004			12.3	<.001			—	—
	Wald H&J sequential			—	—			3.4	.07			.9	.33

*Note:* Number predicted in the different groups and overall proportions of correct predictions for the different tasks. H&J = Hindi and Japanese; writt. = written; phon. = phonological. Correlations which remain significant after Bonferroni correction are in bold.

Group classification is predicted best by written learning, followed by the phonological factor, followed by the H&J sequential task (75%, 71%, and 67% of correct discriminations, respectively). As expected, when written learning and the phonological factor are considered together, discrimination improved to 83% correct classifications. Adding the sequential task to the phonological factor slightly improved prediction (76%), but adding it to written learning had no consequence. These results are consistent with a common order-encoding factor shared by the sequential H&J and written learning. The order-reconstruction task shows weaker associations with orthographic tasks and a less good ability to discriminate dyslexics from controls. However, phonological tasks and written learning involve the same phonological and orthographic representations as those used by reading and spelling. Instead, the order-reconstruction tasks involve completely different representations such as strings of Hindi and Japanese symbols. Thus, weaker associations are not surprising.

## General Discussion

We wanted to assess the contribution of order-encoding deficits to developmental dyslexia. We have measured order encoding with a task that involved reconstructing the order of a number of visually presented, nonalphanumeric characters, immediately after presentation. We administered this task to a large group of adult dyslexics and matched controls along with other tasks that are generally performed poorly by developmental dyslexics and may involve order encoding. As predicted by previous results with a single case study (Romani et al., 1999), the dyslexics were impaired in the order-reconstruction task when presentation of the characters was sequential, but not when they were presented simultaneously. This is consistent with a deficit in the encoding of *temporal* order and with the fact that visuospatial processing, including encoding of spatial order, is generally normal in dyslexic participants (see also Hawelka & Wimmer, 2008; Shovman & Ahissar, 2006; Von Karolyi et al., 2003). Consistent with the hypothesis that order encoding is important for orthographic proficiency, there were extensive correlations between temporal order-encoding and orthographic tasks when the whole group of participants was considered together. The correlations with word and nonword spelling tasks were particularly strong. These correlations were reduced when the groups were considered separately, as one would expect due to a reduced range of abilities and reduced variability, but the correlations with nonword spelling remained significant. This is interesting because we have argued that this task is particularly dependent on a representation of temporal order. Studies with other samples of participants should confirm this specific association.

Ramus and Ahissar (2012) have argued that results are most informative when an impairment in one condition contrasts with intact performance in another. We have achieved this contrast with a careful design and have been able to rule out a number of alternative explanations for our results (see section on Encoding Serial Order). Here, we focus on the nature of a possible order-encoding deficit and its relation with other skills also impaired in dyslexia.

### Specificity of the order-encoding deficit

Our results reveal a deficit in encoding the relative order of stimuli rather than distinguishing between them since no recognition impairment was detected (see also the distinction between “order thresholds”, referring to the ability to detect the order of two stimuli, versus “fusion thresholds”, referring to the ability to detect separate stimuli, made by Ben-Artzi, Fostick, & Babkoff, 2005). We have described this deficit as one involving encoding *temporal order* because dyslexics were impaired in the sequential condition of the task, which requires linking stimuli to positions in time, but not in the simultaneous condition, which requires encoding of spatial positions. It is to be noted, however, that performance on the simultaneous conditions can be spared, not because encoding spatial order is fine, but because visuospatial memory is more able to compensate for an impairment in this condition.

Our results show a striking similarity with others obtained with very different paradigms. It has been found that dyslexics are impaired in comparing dynamic visual stimuli (patches of flickering lines, flickering sinusoidal gratings) only when the stimuli are presented *sequentially*, one after the other, but not when they are presented *simultaneously*, next to one another (Ben-Yehudah & Ahissar, 2004; Ben-Yehudah, Sackett, Malchi-Ginzberg, & Ahissar, 2001; Ram-Tsur, Faust, & Zivotofsky, 2006; Ram-Tsur et al., 2008). We would argue that the sequential conditions of these tasks require a high level of temporal order encoding because one has to remember which one of a series of repeated displays was presented last. Therefore, performance depends on constantly updating information in STM on the basis of the temporal order of the stimuli. There is no need to encode serial order in the simultaneous condition. Consistent with this interpretation, Ram-Tsur et al. (2008) found that the dyslexics performed even worse when the task involved deciding which one out of three sequentially presented displays differed from the other two.^4^

4Like ours, their results are inconsistent with alternative interpretations. SAS and a reduction in temporal resolution predict *better* performance at longer stimulus onset asynchrony (SOA). A memory impairment predicts *worse* performance at longer SOA since information has more time to decay. A difficulty in encoding temporal order, instead, predicts poor performance independent of duration of the stimuli and SOA, and this is what was found (Ram-Tsur et al., 2006, 2008).


### Relation with other tasks

We have not only documented an impairment in encoding temporal order, but also assessed its relation with other impairments that are present in developmental dyslexia and are related to processing temporal order, such as deficits in STM, phonological processing, visual attention, and lexical learning (see introduction). We have found extensive correlations among tasks tapping these skills in the whole group of participants taken together and in the two groups taken separately. Correlations between the Hindi & Japanese sequential task and written lexical learning were particularly high. This is noteworthy since these two tasks superficially have little in common. Szmalec et al. (2011) have argued that dyslexics suffer from a deficit in *long-term order encoding*. Our results, where a severe impairment in lexical learning strongly correlates with an order reconstruction task, support this position.

In a separate study with a subset of our participants, we also found an impairment in allocation of visual attention (conceptualized as a difficulty in splitting attention), and performance correlated with the sequential order reconstruction task described here both in the whole group and in the dyslexics (Pearson *R* range = 45–47, *p* < .01; see Romani, Galluzzi, & Olson, 2011). Consistent with those results, in the simultaneous condition of the order-reconstruction task, dyslexic participants did make more errors at central locations—which are more difficult to discriminate. All of these impairments, including a particularly severe difficulty in nonword reading, are consistent with a deficit in deploying attention to encode serial order.

We find it striking that all the impairments demonstrated by our dyslexics (in the learning, visuoattentional, and phonological domains) have to do with serial order. It is tempting to try to reduce them to a single impairment. However, these skills are not homogeneously impaired across participants, and they show different patterns of correlations with orthographic tasks. Phonological tasks—tapping the quality of phonological representations—were most strongly associated with nonword reading. Written lexical learning—tapping abstract order—was most strongly associated with tasks requiring lexical retrieval such as word reading accuracy and, particularly, word spelling. The sequential condition of the H&J task—tapping temporal order—was most strongly associated with word and nonword spelling. These different patterns of association indicate a degree of independence between these skills. To reconcile these findings we have suggested that one should talk of a family of order-encoding difficulties rather than a single difficulty in processing order.

We believe that STM, phonological processing, visual attention, and lexical learning are all skills that contribute in various degrees to reading and spelling, support different aspects of orthographic processing, and may be impaired in various combinations in different individuals (see Menghini et al., 2010; Ziegler et al., 2008; but also Ramus et al., 2003; Romani et al., 2008; Vidyasagar & Pammer, 2009; White et al., 2006). In addition, however, it is possible that these skills are not completely independent, but share a common core that has to do with order encoding. For example, Bonato, Zorzi, and Umiltà (2012) have recently argued for a space-based mental time line that would be used to order events that enfold in time and space (days of the week, months of the year, numbers, but also, possibly, letters in a word). One possibility is that order can be encoded in a variety of ways—spatially for visual representations, through a phonological record for verbal representations—but that a time-line representation is an important supporting means for these different modalities and, possibly, the preferred means for long-term lexical representations. This will explain why some dyslexics can be selectively impaired in phonology, others in visual processing, and still others in temporal processing (e.g., with lexical learning and nonwords spelling being particularly reliant on a time line), but also why some dyslexics are impaired across tasks tapping order: Deficits will interact, and mild difficulties in processing phonology or in visuospatial attention will be exacerbated by difficulties with a time-line representation and vice versa. Thus, dyslexia will not be caused by damage to a single order-encoding mechanism, but by a family of disorders involving order encoding. Some skills are modality-specific but they could also be supported by a common time-based component.

To detail how damage to different ordering mechanisms may interact in computational models is beyond the scope of this paper. The importance of order-encoding mechanisms has been recognized both in reading and in spelling models. One can note, however, that current models of reading represent letter order with spatially based mechanisms (see the CDP model or the dual-route cascaded model; for a review see Perry, Ziegler, & Zorzi, 2007). Instead, models of spelling have incorporated a time-based sequencing mechanism both to represent the order of letters within lexical representations—such as graded activation levels—and to allow outputting them in the right order—such as competitive cueing mechanisms (see Glasspool & Houghton, 2005; Glasspool, Shallice, & Cipolotti, 2006; Ward & Romani, 1998; see also Vousden, Brown, & Harley, 2000, for the use of time signal to encode order in the phonological lexicon). Ordering mechanisms are more important in the production of written than spoken words for the absence of coarticulation constraints in the written modality. Moreover, ordering mechanisms have been recognized to be especially important for spelling nonwords, which do not receive supporting input from established lexical representations (e.g., see Glasspool et al., 2006). Damage to these mechanisms is broadly consistent with the temporal order-encoding deficit reported here.

If the view outlined above is right, future research should focus on disentangling the unique and shared contribution of different skills to developmental dyslexia. This is important because we need to understand how impairments may exacerbate each other or, alternatively, how preserved skills can be used in compensation. We have provided a good example of this by showing that lexical learning makes a very strong contribution to the severity of dyslexia, but has little impact in control participants. This could be for two complementary reasons: Learning a new word requires learning a new ordering of letters, but also allows for compensation, which is not allowed by other skills. Dyslexics show variation in learning skills. Dyslexic participants with milder deficits may be those who have been able to compensate for their encoding difficulties by developing good lexical representations. This requires good learning skills. Instead, in the controls—consisting mostly of university students, where learning skills are more uniformly high—peripheral encoding skills predict more variation. Thus, correlations between the order-reconstruction tasks and orthographic tasks were stronger in the controls.

The methodological implication of our position is that we need more studies like ours where the same group of individuals is tested with an extensive number of tasks. This is has been done so far only by a relatively small number of studies (see Menghini et al., 2010, for making the same point). Moreover, we need correlations to be carried out separately for controls and dyslexic participants, rather than for the whole group together, as is often done in the literature. As we have demonstrated, when a skill is impaired, compensatory mechanisms can induce stronger reliance on alternative skills, which may determine different correlation patterns in dyslexic and control participants.

### Conclusions

We have been able to demonstrate that a substantial number of adults with developmental dyslexia suffer from an impairment of temporal order encoding and exclude alternative accounts. This result is important since it validates the intuition of many parents, teachers, and clinicians that some children with developmental dyslexia have problems in this domain. However, severe deficits of temporal order encoding are present only in some individuals with dyslexia while others show deficits of phonological processing, long-term order encoding (lexical learning) and visual attention. Moreover, the patterns of association between these deficits and orthographic tasks are different, consistent with independent deficits. We have suggested that “encoding temporal order” is neither “the” skills impaired in dyslexia nor just a skill impaired among others. Instead, we suggest that a representation of serial order is at a core of reading and writing and is supported by a family of skills that contribute to order encoding in different ways. Following this view, an important task for the future is not to find “the” deficit characterizing developmental dyslexia, but to disentangle the relative contribution of different cognitive skills to reading and spelling. This in turn, will allow us to understand which compensatory strategies may be used by individuals with dyslexia to circumvent their cognitive weaknesses.

## Figures and Tables

**Figure A1:**
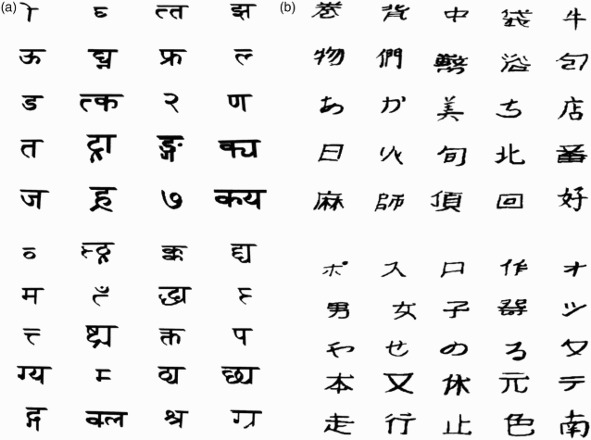
Examples of Hindi and Japanese characters used for the order reconstruction and recognition tasks.
